# Study of the structural characteristics, optical properties, and electrical conductivity of doped [P(An-MMa)/ZrO_2_]^TF^ nanofiber composite using experimental data and TD-DFT/DMol^3^ computations

**DOI:** 10.1007/s11356-022-22477-z

**Published:** 2022-08-22

**Authors:** El-Refaie kenawy, Ali Ibrahim, Ahmed F. Al-Hossainy

**Affiliations:** 1 Polymer Research Group, Chemistry Department, Faculty of Science, Tanta University, Tanta, 31527 Egypt; 2 Physics Department, Faculty of Science, Tanta University, Tanta, 31527 Egypt; 3 Chemistry Department, Faculty of Science, New Valley University, El-Kharga 72511, New Valley, Egypt

**Keywords:** Characterization, Nanofiber composite, TD-DFT/DMol^3^, CASTEP calculations, Optical properties

## Abstract

The powder form of the new nanofiber composite of poly(acrylonitrile-co-methylmethacrylate) (P(An-MMa)) with zirconium dioxide (ZrO_2_) was synthesized using the sol–gel method and subsequently converted to a thin film [P(An-MMa)/ZrO_2_]^TF^ via the physical vapor deposition (PVD) technique. Numerous characterization techniques, including Raman spectroscopy, X-ray diffraction (XRD), thermogravimetric analysis (TGA), scanning electron microscopy (SEM), and ultraviolet–visible (UV–Vis) optical spectroscopy, were used to characterize [P(An-MMa)/ZrO_2_]^TF^. Additionally, using density functional theory (DFT), optimization via time-dependent density functional theory (TD-DFT/DMol^3^) and Cambridge Serial Total Energy Bundle (TD-DFT/CASTEP) was developed. The TD-DFT calculations accurately matched the observed XRD and Raman spectra and validated the molecular structure of the examined materials. The average crystallite size of [P(An-MMa)/ZrO_2_]^TF^, as determined by XRD calculations, is 171.04 nm. The SEM image depicts a one-dimensional morphological structure made up of tightly packed fibrous nanowires or brushes. The optical properties of the films were determined using optical absorbance spectrophotometric results in the 200–850-nm wavelength range. The optical energy bandgaps computed using Tauc’s equation for [P(An-MMa)/ZrO_2_]^TF^ are 2.352 and 2.253 eV, respectively, whereas the isolated molecule of the composite [P(An-MMa)/ZrO_2_]^Iso^ has a bandgap of 2.415 eV as determined by TD-DFT/DMol^3^. The optical characteristics predicted by CASTEP in TD-DFT are in good agreement with the experimental values. The investigated large optical energy bandgap nanofiber composite is advantageous for some energy storage applications.

## Introduction

Polymeric blend and nanocomposite syntheses have lately gained considerable global interest as a growing low-cost approach. The use of nanoparticles in trace levels allows for the efficient development of electrically conductive networks within the insulating polymer. This is in addition to the material’s thermal and optical qualities being improved (Mansour et al. 2022a; Botsi et al. 2019). In order to provide high-power devices, conductive polymer nanocomposite layers of various thicknesses are used to create heterojunction devices and polymer solar cells (Ahmad and Hasan 2015; Shalaby et al. 2022a). In comparison, due to their high refractive and dielectric indices, these items are used in the manufacture of optical equipment and dielectric industrial goods such as condensers (Hassan et al. 2021; Halium et al. 2022). Recently, utilizing PEG200 as a soft template at a pH of 0.65/25 °, a copolymer of different monomers was synthesized through oxidative polymerization, as Zoromba and Al-Hossainy previously described (Zoromba and Al-Hossainy 2020; Abd El-Aal et al. 2022). The extracted copolymer fibers are characterized using a variety of techniques, including FT-IR, proton nuclear magnetic resonance (^1^HNMR), XRD, SEM, and atomic force microscopy (AFM) (Mahmoud et al. 2020; Sain et al. 2013). In the o-toluidine copolymer, the hypochromic shift of [PoPDApT] from 550 to 450 nm implies a reduction in the conjugation range and an increase in the bandgap. The copolymer thin film [PoPDApT]^TF^ is measured to have a thickness of 175 ± 5 nm, where the o-isomers of [PoPDApT] exhibit a greater conductivity than the other isomers. The fact that o-toluidine copolymers have greater conductivity than p-toluidine copolymers indicates that p-toluidine is not a strong enough polymer to be used in the production of o-phenylenediamine copolymers. The optical characteristics and *I*–*V* curves of the Au/n-POPDAPT/p-Si/Al heterojunction device are investigated for varied illumination intensities (Samanta et al. 2015; El Azab et al. 2021a).

Advances and key technologies in the synthesis of different kinds of nanomaterials cover recent advances in the synthesis of various types of nanomaterials. The estimated power conversion efficiency (*PCE*) of Au/n-[PoPDApT]^TF^/p-Si/Al polymer solar cells is around 6.17% at higher light intensities (75 W/m^2^) (Bhagyaraj et al. 2018; Patzke et al. 2011). The nanoparticle size of [ZrO_2_]^NPs^, modified by multiwalled carbon nanotubes (MMC)^NT^ and/or TiO_2_-doped graphite thin film, has lately acquired popularity due to their high melting point, reduced corrosion risk, non-toxicity, and good biocompatibility (Mansour et al. 2022b; Zwawi et al. 2021; Eid et al. 2021). Nanoparticle-sized [ZrO_2_]^NPs^ have a large surface area, making them more efficient in wastewater treatment. Ceramic coatings, construction materials, optoelectronic components, transparent fillers, corrosion inhibitors, and biosensors are made electrochemically (Majeed et al. 2020; Shahnazi et al. 2020). The thermal evaporation technique is employed to create [ZrO_2_]^NPs^ for a nanostructured thin film. The problems of physical vapor deposition (PVD) of high–molar mass organic compounds are important for those creating novel organic electrical devices and materials. Organic electronic material must be heated to the point where it can sublimate and then be deposited at a high rate (0.05–0.2 nm/s) onto the target substrate without thermal deterioration in the crucible or during the gas phase transition between the crucible and the target substrate (Chen et al. 2014; Hrubesh and Pekala 1994). Since Zr(NO_3_)_4_ to rosemary extract of a ratio (1:4) yielded a semi-sphere shape with a mean particle size of 12–17 nm (1:4) (Sajid et al. 2018). We created [ZrO_2_]^NPs^ nanostructured films with a homogeneous structure and surface using a sol–gel approach (Almutlaq and Al-Hossainy 2021; Hu et al. 2020).

The current work reviews contemporary time-dependent density functional theory (TD-DFT) applications (DMol^3^ and CASTEP methodologies) for studying polymer matrix structures, copolymer phase stability, and nanocomposite compounds (Sayyah et al. 2015). This comprehensive energy-based technique has received less attention for its application to the estimate and study of spectroscopic characteristics. The following review examines the estimate of vibrational infrared spectra, XRD, linear and non-linear optical spectra, and spectroscopic difficulties using a restricted programming language (El Azab et al. 2021b). The goal is to show that the same atomistic modeling methodologies may be used consistently throughout the experimental investigation to attain high levels of accuracy (Hammerschmidt et al. 2008). In either standard-conserving or ultrasoft formulations, the potential of an electron ion is expressed using ab initio pseudo-potentials. The results of direct energy minimization are used to obtain the corresponding charge intensity, conscience-consistent technique, and Kohn–Sham wave functions. Techniques for conjugate gradients and density mixing are employed specifically. A strong DFT electron defines a form that may be used to depict systems with a finite number of occupants (Szlachcic et al. 2020). The base set size provided by the energy cutoff for plane waves and the Brillouin zone integration precision calculated using the various *k*-point values used for copolymer and composite compounds are the only two parameters that affect measurement convergence.

In order to enhance the optical and electrical characteristics of the poly(acrylonitrile-co-methylmethacrylate) (P(An-MMa)) copolymer, the current study is focused on evaluating the impact of adding zirconium oxide nanoparticles [ZrO_2_]^NPs^ on the physical properties of the copolymer. The structural and optical properties of the produced nanocomposite thin films were investigated using a new hybrid nanocomposite synthesis [P(An-MMa)/ZrO_2_]^TF^. FT-IR, SEM, and molecular electrostatic potential (MEP) methods can be used to investigate the molecular structure of [P(An-MMa)/ZrO_2_]^TF^. The configuration and polymorph description of [P(An-MMa)/ZrO_2_]^TF^ will also be investigated using XRD spectroscopy. Finally, [P(An-MMa)/ZrO_2_]^TF^ films’ optical and electrical properties were integrated and analyzed. Simulated optical property measurements were also carried out using CASTEP in the TD-DFT technique for [P(An-MMa)/ZrO_2_]^TF^ as-deposited films.

## Experimental

### Materials and reagents

Organic solvents (dimethyl sulfoxide (DMSO), anhydrous dimethylformamide (DMF), diethyl ether, and ethanol), as well as acids, were given by Sigma-Aldrich. Zirconium (IV) nitrate (Zr(NO_3_)_4_, molecular weight = 339.24, 514,535, Sigma-Aldrich), methyl methacrylate (linear formula: CH_2_ = C(CH_3_)COOCH_3_, molecular weight = 100.121 g/mol, M55909, Sigma-Aldrich), and acrylonitrile (linear formula: CH_2_ = CHCN, molecular weight: 53.06 g/mol, Beilstein No. 605310, Sigma-Aldrich). Typically, Si (100) or Si (111) wafers (Sigma-Aldrich) were utilized, where (100) and (111) refer to Miller indices and tolerance. The single crystal of a (p-Si) wafer has a thickness of less than 1 mm and a diameter of 300 mm.

### Synthesis of copolymer P(An-MMa)

**Fig. 1:**
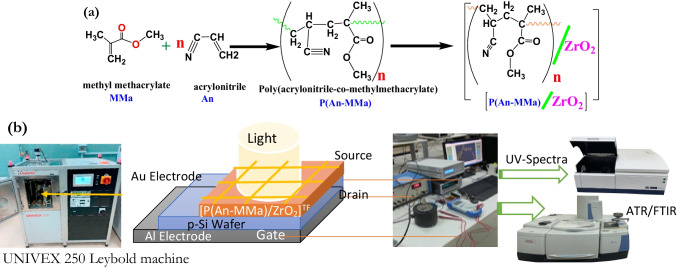
**a** Synthesis of the composite [P(An-MMa)/ZrO_2_]^TF^. (**b**) The schematic diagram of the fabrication of the Au/[P(An-MMa)/ZrO_2_]^TF^/p-Si/Al heterojunction

Poly P(An-MMa) polymer was first synthesized by precipitation polymerization. A solution of 70% distilled water to 30% ethanol was used as the reaction solution. In a ratio of 10% of the total volume of the monomers and polymerization solution, a 1:1 combination of methyl methacrylate (MMa) and acrylonitrile (An) monomers was added to the polymerization solution. Potassium persulfate (K_2_S_2_O_8_) was added to the mixture as a reaction initiator by 0.05 M. A hot plate with a magnetic stirrer was used to hold the polymerization process for 4 h at 55 °C. An ethanol–distilled water mixture was used to wash the final product after it had been centrifuged and then lifted to dry for 24 h at 55 °C (Fig. 1a). The molecular weight of P(An-MMa) was measured using the static light scattering technique and found to be 9.7 × 10^5^ g/mol (Ghazy et al. 1999).

### Synthesis of [P(An-MMa)/ZrO_2_]^TF^ and fabrication of Au/[P(An-MMa)/ZrO_2_]^TF^/p-Si/Al heterojunction derivative

PVD was employed to make [P(An-MMa)/ZrO_2_]^TF^ thin films. Using a UNIVEX 250 (Leybold, Germany) at a base pressure of 5 × 10^3^ Pa, interdigitated electrodes separated by 75 μm, and a deposition rate of 3 Å/s, thin films were deposited onto a single crystal of a (p-Si) wafer and an ITO/glass substrate. The W boat tantalum was deposited at a rate of 2–3 Åm/s through a shadow mask to create a 110-μm-long channel with a 1.2-mm-wide channel. The nanostructure [P(An-MMa)/ZrO_2_]^TF^ thin films had a thickness of 98.30 nm and were balanced on a UNIVEX 250 Leybold machine utilizing a quartz crystal microbalance (as shown in Fig. 1b). The vacuum pressure used in this investigation was 5 × 10^3^ Pa, compared to 27 × 10^2^ and 27 × 10^1^ Pa in earlier studies (Lee et al. 2013; Mahmoud et al. 2020). From the substrate contact to the film surface, no compositional alterations suggestive of reactivity with the substrate were found. Additionally, based on the finding, the change in vacuum pressure was not used to construct [P(An-MMa)/ZrO_2_]^TF^ nanofiber composite thin films.

### A computational study for [P(An-MMa)/ZrO_2_]^Iso^ as gaseous isolated molecules

The effectiveness of frequency dimensions and molecular structure for [P(An-MMa)/ZrO_2_]^Iso^ in the gas phase was estimated using CASTEP and DMol^3^ calculations according to TD-DFT/CASTEP and TD-DFT/DMol^3^ calculations, respectively. For free molecules, DMol^3^ software and CASTEP software were used to determine the Perdew–Burke–Ernzerhof (PBE) exchange, general gradient approximation (GGA) functional correlations, the DNP base set, and the pseudo-conserving norm (Becke 1992; Miehlich et al. 1989). In the structural matrix simulation computations, the plane-wave cutoff energy was set to 550 eV. Using DMol^3^ and CASTEP frequency computation findings at the gamma point (GP), the optical characteristics, structural or spectroscopic features, and XRD patterns of [P(An-MMa)/ZrO_2_]^Iso^ were investigated. Using the functional Becke’s non-local interchange, the functional B3LYP of [P(An-MMa)/ZrO_2_]^Iso^ in the gas phase was determined (Frisch et al. 2009). IR vibrational frequency measurements were carried out using WBX97XD/6-311G. The vibration modes, geometric properties, energy, and optimal structure visualization were studied using the GAUSSIAN 09 W software system (Al-Hossainy et al. 2018). DFT calculations based on WBX97XD/6-311G when using the B3LYP method, as shown in previous work, have produced excellent findings for structural spectrum correlation, including numerous important experimental discoveries (Thabet et al. 2020). The Gaussian potential approximation system (GAP) specifies the concurrent use of several independent uncertainty models and the overall power and derivative model to assign Gaussian and CASTEP [P(An-MMa)/ZrO_2_]^Iso^ models in the gas phase.

### Characterizations

Table 1 shows how [P(An-MMa)/ZrO_2_]^TF^ is distinguished by the analytical techniques and circumstances.

**Table 1:** Different analytical techniques and measurement conditions

Techniques	Instruments
TGA and Dr TGA	At an accuracy of 0.1 K, simultaneous TGA/DrTGA analyses were performed using a DTG-50H (Shimadzu, Japan) calorimeter in a temperature range from 40 to 700 °C
Raman spectroscopy	Using PerkinElmer FT-IR type 1650 spectrophotometer in the wave number range 4000–400 cm^−1^, in which the spectra were recorded through KBr pellets
XRD	PANalytical X’Pert PRO (Shimadzu, Holland) apparatus with a nickel filter and a Cu Kα X-ray target (located in Central Lab, Tanta University, Tanta, Egypt). Scanning range 4–90°, step side 0.02, scan rate 1 s, and anode supply copper are the conditions for condition 2*θ*
UV–Vis	Using a PerkinElmer Lambda 365 spectrophotometer, the UV–Vis spectra of both [P(An-MMa)]TF copolymer and [P(An-MMa)/ZrO_2_]^TF^ nanofiber composite films were measured in the range of $200 \mathrm{nm}\le \lambda \le 850 \mathrm{nm}$
Electrical conductivity	Keithley 6517B/with 2 Au pads was sputtered into the films to measure the electrical conductivity of the [P(An-MMa)/ZrO_2_]^TF^ nanofiber composite thin film. The film metrics for the [P(An-MMa)/ZrO_2_]^TF^ nanofiber composite film have been measured at various temperatures in an ambient environment without packaging

## Results and discussion

### Thin film (experimental) and TD-DFT (simulated) Raman spectroscopy

**Fig. 2:**
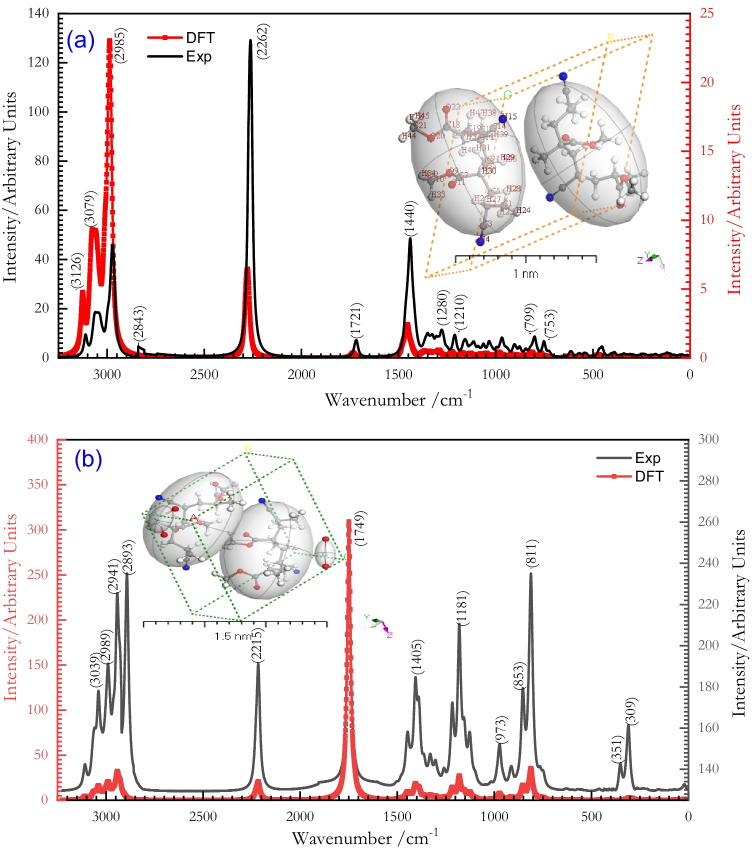
**a** Experimental and simulated Raman spectra of thin film [P(An-MMa)]^TF^ and the structure of P(An-MMa) obtained from *Gaussian 09 W software* with DFT/WB97XD and basis set 6-311G. (**b**) Experimental and simulated Raman spectra of [P(An-MMa)/ZrO_2_]^TF^ and the structure of [P(An-MMa)/ZrO_2_]^Iso^ obtained from *Gaussian 09 W software* with DFT/WB97XD and basis set 6-311G

Raman spectroscopy reveals the chemical structure, crystallinity, and molecular interactions of the samples under investigation. It is based on light’s interaction with a material’s chemical bonds. The experimental Raman spectra of both [P(An-MMa)]^TF^ copolymers and [P(An-MMa)/ZrO_2_]^TF^ nanofiber composite thin films are presented in Fig. 2a, b. Raman spectroscopy of the [P(An-MMa)]^TF^ copolymers showed all the characteristic peaks (Fig. 2a), where each peak corresponds to a specific molecular bond vibration. Seven main peaks with different intensities and wave numbers (cm^−1^) were obtained. These peaks had a different value of wave number as 799, 1440, 1721, 2262, 2985, 3079, and 3126 cm^−1^. After the consecutive increase in the loading of ZrO_2_ nanoparticles in [P(An-MMa)]^TF^ copolymer to form the [P(An-MMa)/ZrO_2_]^TF^ nanocomposite as shown in Fig. 2b, fourteen main characteristic peaks appeared. Also, all peaks were characterized by a higher band intensity in comparison to those for the copolymer only. Approximately seven new peaks of wave numbers at 309, 351, 853, 973, 1181, 2893, and 2941 cm^−1^ were obtained. Peaks display a little shift change in the band’s peak location, which may result from secondary bonding in nanofiber composites between [P(An-co-MMa)] and [ZrO_2_]^NPs^. Or, band shifts in values and position represent the disorder developed in sp^2^ carbon atoms due to the incorporation of [ZrO_2_]^NPs^ with copolymers (Shalaby et al. 2022b; Ribeiro et al. 2015).

The spectroscopic peaks of the isolated [P(An-MMa)/ZrO_2_]^Iso^ molecule in the gaseous state were determined using the theoretical Raman spectrum. The inset figure in Fig. 2 depicts the slight differences between the expected and evaluated frequencies. The main distinction is that the count was done in a vacuum, whereas the calculations were done in a solid state. Because the ligands analyzed have complicated vibrational modes, they contribute to low symmetry, making it difficult to ascribe the torsion as well as the plane modes because ring modes deteriorate alongside imitative ones. The graph achieved, however, shows some apparent fluctuations. The direct correlation among the calculated wave numbers (${\upsilon }_{Cal}.$) for [P(An-MMa)/ZrO_2_]^Iso^ and experimental wave numbers (${\upsilon }_{Exp}.$) for [P(An-MMa)/ZrO_2_]^TF^ is illustrated by the next equation: ${\upsilon }_{Cal}. =0.89{\upsilon }_{Exp}. +37.91$ with correlation coefficient (${R}_{2}=0.927$) (Shalaby et al. 2022a; Preefer 2020).

### TGA and DrTGA thermal analysis of P(An-MMa) and [P(An-MMa)/ZrO_2_]

**Fig. 3:**
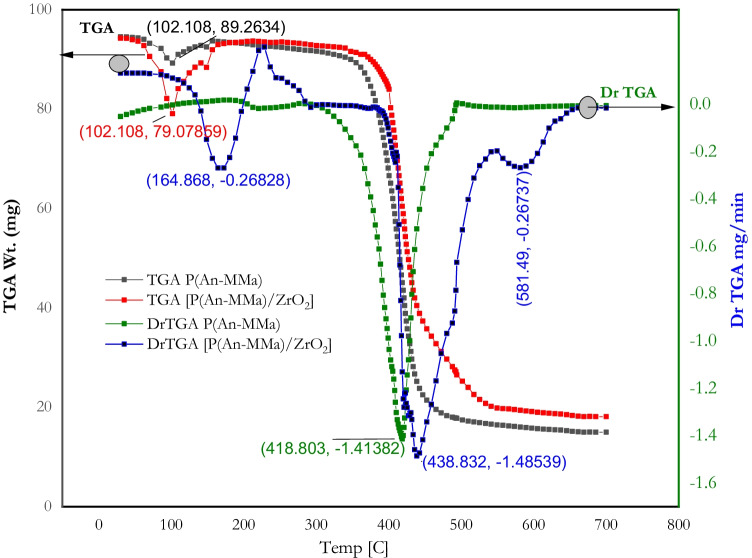
The thermal behavior (TGA and DrTGA) of P(An-MMa) and [P(An-MMa)/ZrO_2_]

A thermogravimetric analyzer (Shimadzu DTG-50H, Japan) was used to investigate the thermal degradation spectra of both [P(An-MMa)] copolymers and [P(An-MMa)/ZrO_2_] nanofiber composite in the temperature range of 20 to 700 °C under a dynamic nitrogen atmosphere at a flow rate of 20 ml/min and a heating rate of 10 °C/min (Fig. 3). From the obtained results of the TGA spectra of both P(An-co-MMa) copolymers and [P(An-MMa)/ZrO_2_] nanofiber composite, the curves can be divided into four steps according to weight loss: (i) in the first one from 20 to 150 °C, a mass loss has been obtained, where at 102.1 °C, a mass loss of 89.26% and 79.078% of both P(An-co-MMa) copolymers and [P(An-MMa)/ZrO_2_] nanofiber composite was obtained, respectively. On the other hand, (ii) from 150 to 375.24 °C, no mass loss and thermal stability of both P(An-co-MMa) copolymers and [P(An-MMa)/ZrO_2_] nanofiber composite were obtained, in comparison to PMMA and PAN, which showed thermal stability up to 265 °C and 269 °C, respectively (Lan and Shi-Chao 2007; Ahmed et al. 2022). An enhancement of the thermal stability by loading the ZrO_2_ nanoparticles to the copolymers by 7–8% has been obtained. (iii) This step was in a temperature range from 375 to almost 450 °C; a sharp weight rate loss occurred because of the dehydrogenation of the copolymers to some extent. In the last step, (iv) the temperature > 550 °C disintegration of polymer chains happened to create volatile particles causing a weight loss; at the same time, the oxygen uptake reactions took place, leading to a certain amount of weight gain through the generation of oxygen-containing groups such as –OH and C $=$ O. Generally, the weight loss in [P(An-MMa)/ZrO_2_] nanofiber composite is less than that of the copolymers P(An-co-MMa). Thus, the total weight loss and weight gain gave expressed weight loss.

Also, Fig. 3 demonstrates the Dr TGA of both P(An-co-MMa) copolymers and [P(An-MMa)/ZrO_2_] nanofiber composite. The weight losses were distinguished with exothermic and endothermic peaks. Regarding P(An-co-MMa) copolymers, they were characterized by an exothermic peak (i.e., up to 330 °C and 500 °C to the end) and also just one endothermic peak at (418.83 °C, − 1.4138 mg/min rate loss) within the temperature range of decomposition. In addition, the nanofiber composite [P(An-MMa)/ZrO_2_] was characterized by a different behavior. Three exothermic peaks were obtained (up to 110 °C, 300–400 °C, and 600 °C to the end), while there were another three endothermic peaks at (164.868 °C, − 0.26828 mg/min), (438.832 °C, − 1.48539 mg/min), and (581.49, − 0.26737 mg/min) within the temperature range 20 to 700 °C of decomposition.

### XRD analysis of °the thin film (experimental) and TD-DFT (simulated)

**Fig. 4:**
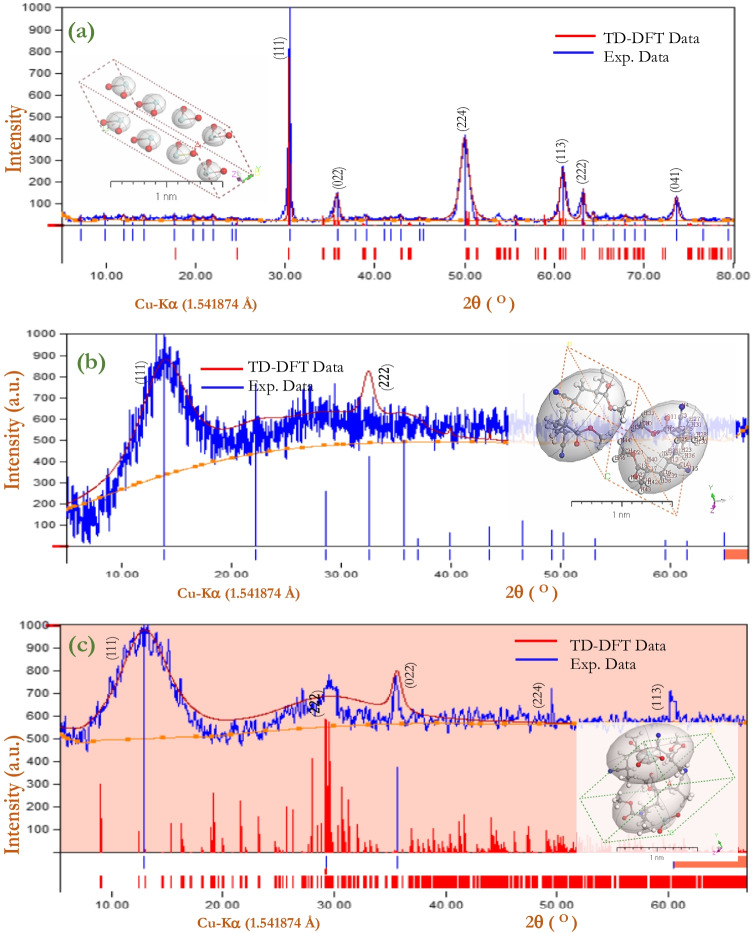
**a** PXRD patterns (combined experimental and simulated) of [ZrO_2_]^TF^ thin film and [ZrO_2_)]^Iso^ isolate molecule. The inset is a 3D *orthorhombic Pbcm (57)* lattice-type computed using the polymorph method. (**b**) [P(An-MMa)]^TF^ and [P(An-MMa)]^Iso^. The inset is a 3D *monoclinic C1c1 (9)* lattice type. (**c**) [P(An-MMa)/ZrO_2_]^TF^ and [P(An-MMa)/ZrO_2_]^Iso^. The inset is a 3D *triclinic P-1 (2)* lattice type

The XRD spectral profile obtained for the fabricated thin film of [P(An-MMa)/ZrO_2_]^TF^ (experimental) is compared with the isolated system matrix (simulated by TD-DFT). The XRD patterns in Fig. 4 demonstrate that [P(An-MMa)/ZrO_2_]^Iso^ and [P(An-MMa)/ZrO_2_]^TF^ are nearly identical. Table 2 shows how the absolute values of the full width at half maximum (FWHM) affect the estimated average crystallite size (*D*) and Miller index (*hkl*). The data in database code_amcsd 96–900-5835 (Kudoh et al. 1986), 96–901-0660 (Demartin et al., 2008), and 96–431-5914 (Li et al. 2010) are in good agreement with the interplanar distances (*d*). The Microsoft apps TDDFT-DFT and Crystal Sleuth produced diffraction peaks that were quite close to the measured data of [ZrO_2_]^NPs^, [P(An-MMa)]^TF^, and [P(An-MMa)/ZrO_2_]^TF^, respectively. The Debye–Scherrer relation is applied to evaluate the XRD pattern of [ZrO_2_]^NPs^, [P(An-MMa)]^TF^, and [P(An-MMa)/ZrO_2_]^TF^, within the range of 5 ≤ 2*θ* ≤ 80° with  ,1/dhkl = 0.0566Å^−1^ − 0.7446Å^−1^, 𝜆 = 1.540562 Å, ${I}_{2}/{I}_{1}=0.5$, polarization nearly equal to 0.5, and pseudo-Voigt function. From Scherer’s formula, $D=0.9\lambda /(\mathrm{FWHM}\bullet \mathrm{cos}\theta )$, where *λ* is the X-ray wavelength (1.541838 Å) (Baghdadi et al. 2021; Clarisse 2013). As presented in Table 2, the fabricated [ZrO_2_]^NPs^, [P(An-MMa)]^TF^, and [P(An-MMa)/ZrO_2_]^TF^ XRD data were used to study factors and features such as FWHM, the average crystallite size (*D*), Miller indices (*hkl*), *d*-spacing (*d*), and peak intensity. The range of crystalline sizes for [ZrO_2_]^NPs^ is 3.579 nm at 2*θ* = 49.92° to 17.04 nm at 2*θ* = 30.45°, whereas the range of crystalline sizes for the blend and nanocomposite is 87.47 nm at 2*θ* = 13.80° to 133.91 nm at 2*θ* = 27.72° and 60.37 nm at 2*θ* = 50.62° to 272.43 nm at 2*θ* = 29.53°, respectively. The average crystalline size is ${D}_{Av}=7.504, 110.69,\mathrm{ and\;}171.04\mathrm{ nm}$ for [ZrO_2_]^NPs^, [P(An-MMa)]^TF^, and [P(An-MMa)/ZrO_2_]^TF^, respectively (Zoromba et al. 2021; Abd-Elmageed et al. 2020; Abrahams et al. 2011). In addition, the polymorph used Content Studio software computations to determine theoretical XRD models (version 7.0) (see Fig. 4 inset). The integrals performed on the Brillouin zone are shown in the inset of Fig. 4 with 2 × 2 × 1 (polymorph [ZrO_2_]^Iso^, [P(An-MMa)]^Iso^, and [P(An-MMa)/ZrO_2_]^Iso^). For the relevant experimental, a comparison was made between XRD (experimental) structures and estimated PXRD patterns. The strength and position of specific peaks differ only slightly between the experimental and simulated XRD models; therefore, the focus here is on their general similarity, since instrumentation and data collection techniques are merely two of the many factors that can influence the experimental XRD pattern (Mohamed et al. 2022).

**Table 2:** The experimental and calculated XRD data using the *Refine version 3.0 software program (Kurt Barthelme and Bob Downs*) for [ZrO_2_]^NPs^, [P(An-MMa)]^TF^, and [P(An-MMa)/ZrO_2_]^TF^

Symmetry	Observed					Computed			
	2*θ* (°)	*d* (Å)	*hkl*	FWHM	Int	2*θ* (°)	*d* (Å)	${D}_{Av}$^(a)^	$\delta$^(b)^
[ZrO_2_]^NPs^	30.45	2.94	*111*	0.2798	1.214	30.55	2.93	17.04	5.88
Orthorhombic Pbcm (57)	35.64	2.52	*022*	0.8249	158.2	35.58	2.52	5.768	17.34
*a* = 5.005, *b* = 5.24, and *c* = 5.05 nm	49.92	1.83	*224*	1.3294	457.2	49.90	1.83	3.579	27.94
*α* = *γ* = *β* = 90°	60.92	1.52	*113*	0.9444	279.3	60.92	1.52	5.038	19.85
*V* = 820 (1); *rmse* = 0.00095427	63.10	1.47	*222*	0.6238	148.2	63.10	1.47	7.627	13.11
	73.57	1.29	*041*	0.7894	117.3	73.58	1.29	6.027	16.59
[P(An-MMa)]^TF^ monoclinic C1c1 (9)								**7.507**	**13.32**
= 15.82; *b* = 9.14, and *c* = 13.75 nm	13.80	6.45	*111*	5.4393	930.2	13.79	6.45	87.47	11.43
*α* = *γ* = 90°, *β* = 112.44°	27.72	3.22	*222*	3.5529	602.3	27.72	3.22	133.91	7.468
*V* = 1393(5); *rmse* = 0.0000002313								**110.69**	**9.034**
[P(An-MMa)/ZrO_2_]^TF^	12.85	6.97	*111*	6.8310	965.3	12.84	6.97	69.65	14.36
Triclinic P-1 (2)	29.53	3.04	*222*	1.7464	750.2	29.53	3.03	272.43	3.671
*a* = 6.88; *b* = 6.89; *c* = 6.93	35.56	2.53	*022*	0.5233	780.7	35.56	2.53	90.92	10.99
*α* = 93.03; *β* = 96.23°, *γ* = 94.38°	60.37	1.54	*113*	0.9399	680.5	60.37	1.53	50.62	19.76
*V* = 804(2); *rmse* = 0.00000028769									
**Average**				**0.7413**				**171.04**	**5.85**

*Int.* intensity, *rmse* root mean square error

^(^^a)^nm

^(^^b)^10^−3^

The simulated XRD of [ZrO_2_]^Iso^, [P(An-MMa)]^Iso^, and [P(An-MMa)/ZrO_2_]^Iso^ as isolated molecules provides *orthorhombic Pbcm (57)*, *monoclinic C1c1 (9)*, and *triclinic P-1 (2)* structures, respectively. For the experimental patterns of [ZrO_2_]^NPs^, [P(An-MMa)]^TF^, and [P(An-MMa)/ZrO_2_]^TF^, the main peaks at *hkl* of (111) are at 2*θ* values of 30.45°, 13.80°, and 12.85°, respectively. The accuracy of the fabricated material PXRD patterns is validated by the above assessment, which shows good agreement between the calculated and experimental PXRD patterns. A combination of the calculated and experimental PXRD patterns yielded a great estimation of the atomic scale of [ZrO_2_]^NPs^, [P(An-MMa)]^TF^, and [P(An-MMa)/ZrO_2_]^TF^ (Mahmoud et al. 2021). The specific surface area ($\varphi$) of [ZrO_2_]^NPs^ is associated with the average crystallite size (*D*) by the equation $\varphi =6/\rho D=1.41{\times }^{-2}{\mathrm{cm}}^{2}$ (Rahman and Nasir 2018), where $\rho$ is the density of [ZrO_2_]^NPs^ (5.68 g/cm^3^). Also, the defect density is calculated by (Ibrahim et al. 2018) $\delta =1/D$ specified as the dislocation line length per unit volume.

### Morphological study of [P(An-MMa)/ZrO_2_]^TF^ nanocomposite thin film

**Fig. 5:**
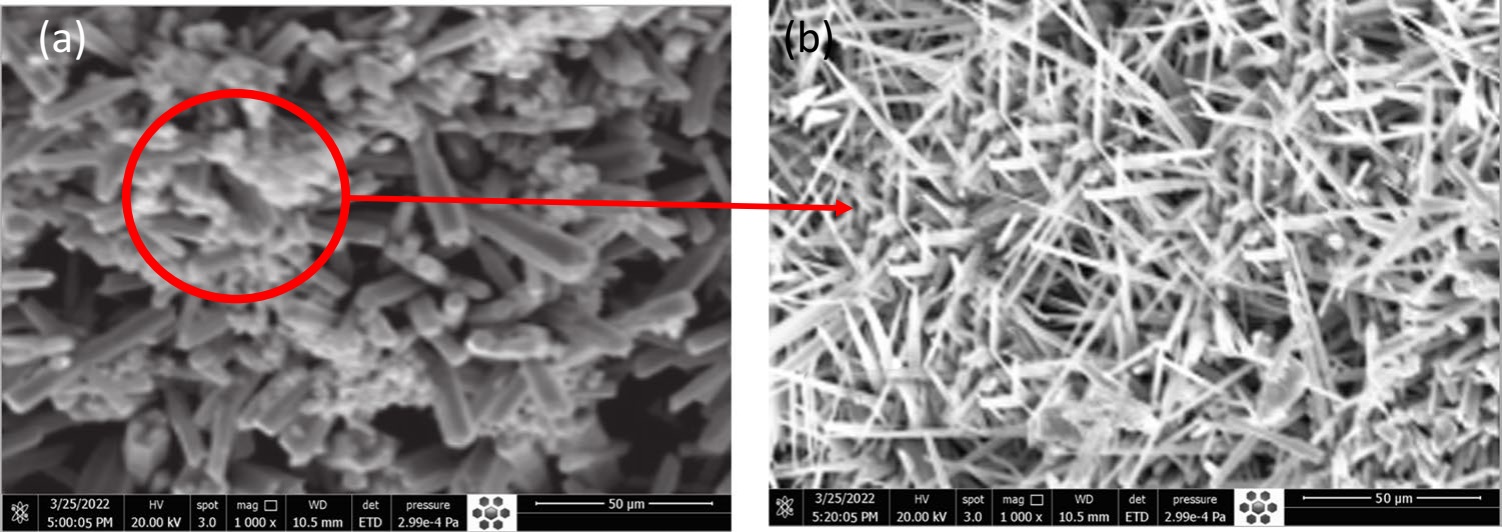
**a**, **b** Top-view SEM images of [P(An-MMa)/ZrO_2_]^TF^

SEM of [P(An-MMa)/ZrO_2_]^TF^ was used to study the various topologies (morphologies), as shown in Fig. 5. The image depicts a one-dimensional morphological structure made up of tightly packed fibrous nanowires or brushes, which is similar to the uniformity of structures generated. Figure 5 demonstrates a long-range similarity (homogeneity) of the nanowires (nanofibers) with an average thickness of ≅ 4.11 × 10^3^ ± 110 nm and a length of ≅ 69.10–41.13 ± 2 μm of [P(An-MMa)/ZrO_2_]^TF^ thin films. The increased surface area is achieved by the fiber structures formed during the manufacture of the nanocomposite powder. [P(An-MMa)/ZrO_2_]^TF^ is synthesized through self-assembly of co-monomers units that have -type bonds. The topology of [P(An-MMa)/ZrO_2_]^TF^ provides clarification for the self-assembling process by including both intermolecular π–π* interactions and hydrogen bonds among copolymer chains (Zoromba et al., 2018).

### Geometric study of [P(An-MMa)/ZrO_2_]^Iso^

**Fig. 6:**
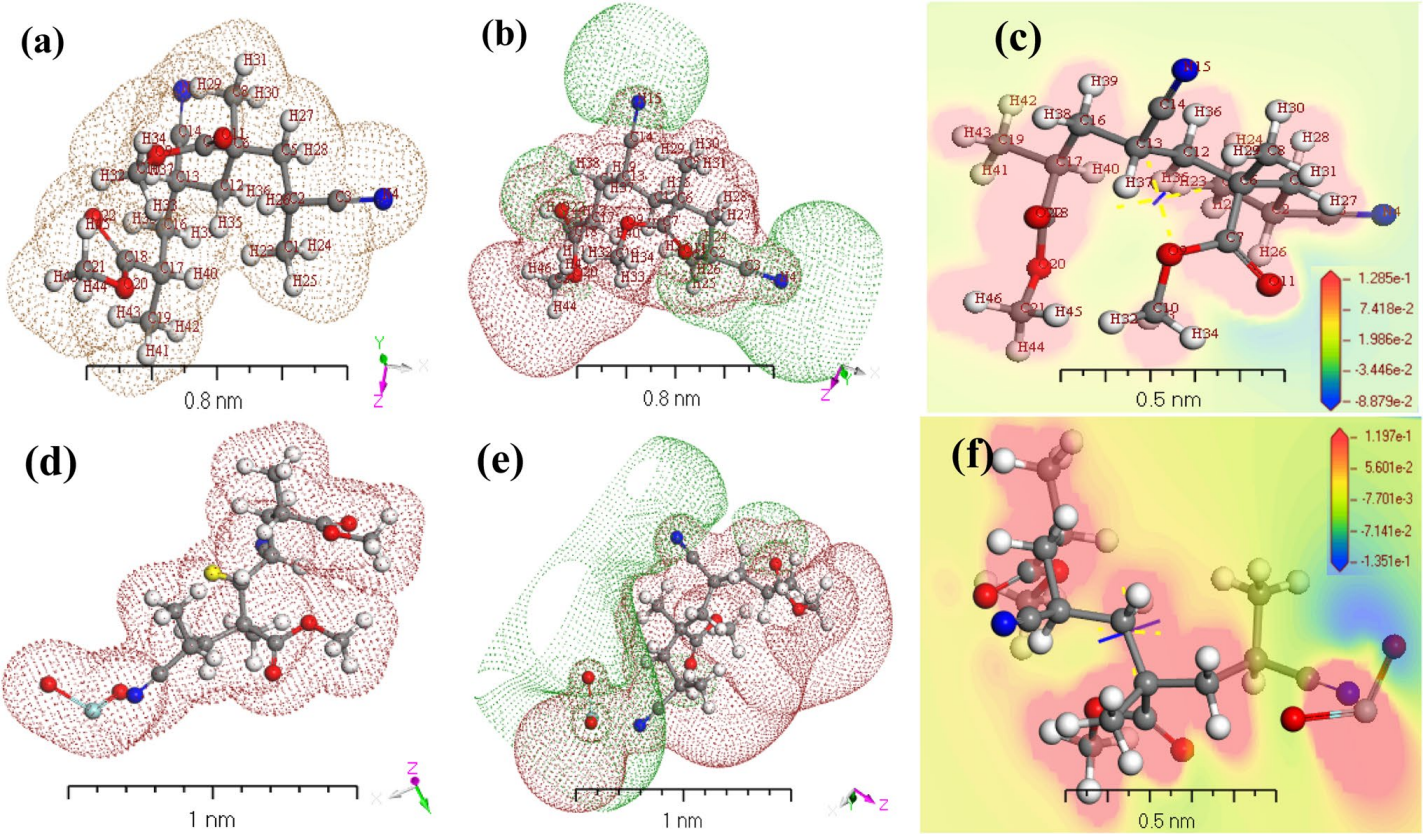
**a**, **d** Electron density of [P(An-MMa)]^Iso^ and [P(An-MMa)/ZrO_2_]^Iso^ gas phase. (**b**, **e**) Potential of [P(An-MMa)]^Iso^ and [P(An-MMa)/ZrO_2_]^Iso^ gas phase and (**c**, **f**) MEP of [P(An-MMa)]^Iso^ and [P(An-MMa)/ZrO_2_]^Iso^ as isolate state utilizing *TD-DFT/DMol*^*3*^ programs

Using electrostatic potential and electron density, the similarity of the gaseous phase physical–chemical properties of [P(An-MMa)/ZrO_2_]^Iso^ was investigated (Reis et al. 2020; Mori-Sanchez et al. 2003) (Fig. 6a–f). While TD-DFT and TD-DFT/Gaussian concepts use electron density as an essential operator for the assessment of the isolated state of [P(An-MMa)/ZrO_2_]^Iso^ gas-phase electron systems (as shown in Fig. 6a and d). The potential diagrams demonstrating significant potential growth of the [P(An-MMa)/ZrO_2_]^Iso^ gas phase were investigated as in Fig. 6b and e. The results support the possibility of electron transfer in [P(An-MMa)/ZrO_2_]^Iso^ in a gas phase to calculate the molecular electrostatic potential (MEP) according to its surface density. Figure 6c and f show 3D images of the active sites of the MEP through the [P(An-MMa)/ZrO_2_]^Iso^ isolated molecules. The blue and red colors demonstrate the areas advantageous for nucleophilic and electrophilic attacks, respectively. In the isolated molecule phase, the MEP range of the [P(An-MMa)]^Iso^ and [P(An-MMa)/ZrO_2_]^Iso^ matrix is − 8.879 × 10^−2^ ≤ [MEP] ≤ 1.285 × 10^−1^ and − 1.35 × 10^−1^ ≤ [MEP] ≤ 1.97 × 10^−1^. The MEP diagram shows potential negative areas of positive potential for hydrogen atoms. The color order was found to be red < brown < blue (Mori-Sanchez et al. 2003; Abed-Elmageed et al. 2020). The strongest electronic attraction is represented by blue, whereas the strongest electronic repulsion is represented by red (Taha et al. 2019; Mohamad et al. 2019). The lone pair of electronegative atoms is aligned with the negative regions. The studied molecule MEP maps discovered that negative regions are concentrated on the chloride, nitrogen, and oxygen molecules. On the other hand, the highly positive regions are located on the deprotonated –O–(CO)–C– groups in the copolymer [P(An-MMa)]^Iso^ and also in ZrO_2_ in the composite, considered a potential nucleophilic attack site with a maximum value of + 3.87 a.u. The computations indicate that the MEP map showed negative potential sites for (–C≡N–) and (–C = O), while it showed positive potential sites for –CH_2_C–. These locations give valuable information about the intermolecular interactions of the molecule. As a result, Fig. 6c and f confirm the occurrence of intermolecular hydrogen bonding.

**Fig. 7:**
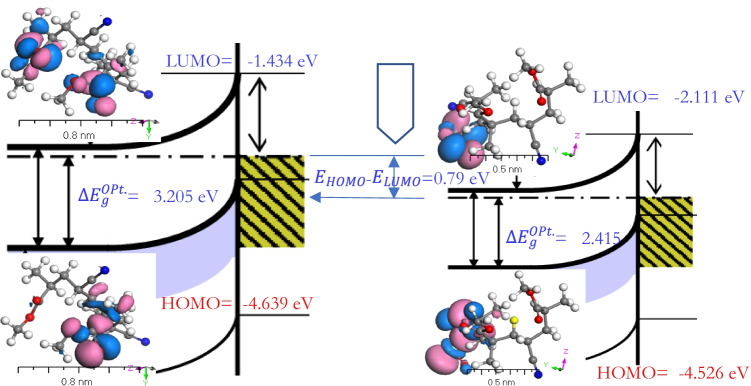
Energy band diagrams of [P(An-MMa)]^Iso^ and [P(An-MMa)/ZrO_2_]^Iso^. All quantities are given in units of eV

The measured $\Delta {E}_{g}^{Opt}$ values were based on the discrepancy between the highest occupied molecular orbital (HOMO) and the lowest unoccupied molecular orbital (LUMO) utilizing the *TD-DFT/DMol*^*3*^ procedure as shown in Fig. 7. HOMO and LUMO are essential parameters in quantum chemical simulations for the complicated analysis known as the fragment molecular orbital method (FMO). The computed energy values ${E}_{\mathrm{HOMO}}$, ${E}_{\mathrm{LUMO}}$, and $\Delta {E}_{g}^{Opt}$ are presented in Table 3. The tabulated values of the chemical potential (*μ*), softness ($\sigma )$, global softness (*S*), global hardness ($\eta )$, electronegativity (*χ*), and global electrophilicity index (*ω*) and the maximum amount of electronic charge (${\Delta N}_{max})$ were calculated using the following equations: $=({E}_{\mathrm{HOMO}}+{E}_{\mathrm{LUMO}})/2,$ $\chi =- \mu$, $\eta ={(E}_{\mathrm{LUMO}}-{E}_{\mathrm{HOMO}})/2$, $S=1/2\eta$, $\omega =\mu 2/2\eta$, $\sigma =1/\eta$, and $\Delta {N}_{max}=-\mu /\eta$ (Abdel-Aziz et al. 2021; Kaya et al. 2016a). The negative values of ${E}_{\mathrm{HOMO}}$ and ${E}_{\mathrm{LUMO}}$ energies can be ascribed to the stability of [P(An-MMa)/ZrO_2_]^Iso^ as an isolated molecule. *ω* is a critical quantum chemical feature where it evaluates the energy stability when the device receives an additional electronic charge (Kaya et al. 2016b).

**Table 3:** Geometry is constant for [P(An-MMa)]^Iso^ and [P(An-MMa)/ZrO_2_]^Iso^ as isolated molecules

Compounds	*E*_HOMO_	*E*_LUMO_	(*E*_H_ − E_L_)	*χ* (eV)	*µ* (eV)	*η* (eV)	*S* (eV)	*ω* (eV)	$\Delta {N}_{max}$	$\sigma$( eV^−1^)
[P(An-MMa)]^Iso^	− 4.639	− 1.434	3.205	3.037	− 3.037	1.603	0.312	2.877	1.895	0.624
[P(An-MMa)/ZrO_2_]^Iso^	− 4.526	− 2.111	2.415	3.319	− 3.319	1.208	0.414	4.560	2.748	0.828

### Optical properties of [P(An-MMa)]^TF^ and [P(An-MMa)/ZrO_2_]^TF^ thin films

**Fig. 8:**
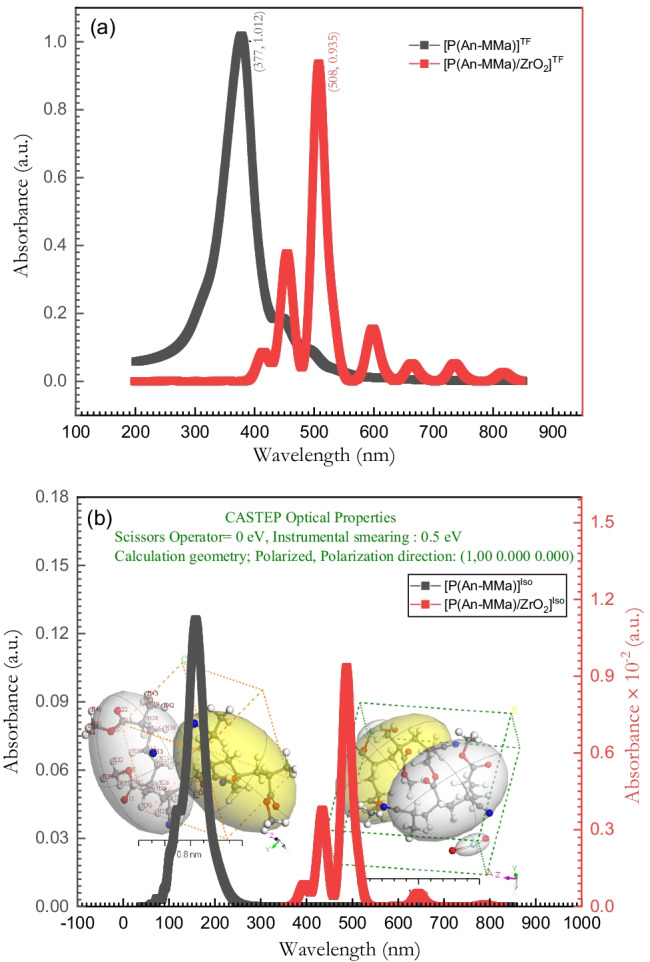
**a** The calculated absorbance experimental data of [P(An-MMa)]^TF^ and [P(An-MMa)/ZrO_2_]^TF^ and (**b**) TD-DFT/CASTEP of [P(An-MMa)]^Iso^ and [P(An-MMa)/ZrO_2_]^Iso^ spectra. The inset figure is a 3D *triclinic* lattice type computed using the polymorph method

Because PMMA is amorphous and belongs to the acrylate family, it has high optical clarity in the visual spectrum of electromagnetic radiation. A copolymer sequence including acrylonitrile (An) in varied weight percent ratios was produced. After that, a loading of ZrO_2_ nanoparticles to the copolymer has been done. The optical properties of [P(An-MMa)]^TF^ copolymer and [P(An-MMa)/ZrO_2_]^TF^ films were examined with a UV–visible spectrophotometer. UV–Vis absorbance spectra of both [P(An-MMa)]^TF^ copolymer and [P(An-MMa)/ZrO_2_]^TF^ nanofiber composite thin films are shown in Fig. 8. The UV–Vis experiment was carried out in absorbance mode and as a function of the wavelength ranging from 200 to 850 nm at room temperature (Fig. 8a). From Fig. 8a, the [P(An-MMa)]^TF^ copolymer film has one sharp absorption band peak at 377 nm. This sharp peak is attributed to the π–π* transition which is related to the degree of conjugation between the polymer chains. On the other hand, Fig. 8a also shows the absorbance spectrum of the [P(An-MMa)/ZrO_2_]^TF^ nanofiber composite film. The nanofiber composite film has a redshift in the peak position. For $394\mathrm{ nm}<\lambda <559 \mathrm{nm}$, there are three peaks, where the main peak is exhibited at 508 nm. The n–π* the transition between the HOMO orbital of the benzenoid and the LUMO orbital of the quinoid rings may be responsible for these absorption peaks (Amarnath et al. 2008; Al-Hossainy et al. 2021). Moreover, four weak band transitions in the range 565–850 nm are located at 600 nm, 665 nm, 736 nm, and 815 nm, respectively. This region of the spectrum signifies the Q-band type. The type and position of substituents on the nanofiber composite rings have a significant impact on the intensity of Q bands. Thus, the signal intensities of the Q bands with $\uppi$-electrons attached directly to the $\mathrm{\alpha }$-positions change. π–π* excitation between bonding and antibonding molecular orbitals provides characterized peaks for the nanofiber composite film in the Q-band region. Due to the opposite direction of the electric dipoles and the cancellation of electric dipoles that occurs, absorption transitions in the Q-band region have tiny oscillator strengths, resulting in low intensity in the Q bands. The main absorption band, on the other hand, shows an intensity that is higher than that of Q bands (Albuquerque et al. 2000; Xie et al. 2001). Figure 8b reveals the simulation absorption spectra by using the CASTEP method in DFT. In most of the wavelength spectra investigated, especially for the nanofiber composite film, the experimental approach and DFT predictions for the absorbance spectra show a good similarity. On the other hand, the copolymer film exhibits an ultraviolet shift.

### Energy gap calculation of [P(An-MMa)]^TF^ copolymer and [P(An-MMa)/ZrO_2_]^TF^ nanofiber composite thin film

Near the fundamental absorption band edge, both direct and indirect transitions arise and can be detected by plotting ${\alpha }^{2}$ and ${\alpha }^{0.5}$ as a function of the photon energy ($h\nu$) (Davis and Shilliday 1960). The analysis of both allowed transitions is based on the following relations (Thutupalli and Tomlin 1976):

1 $${\left(n\alpha h\nu \right)}^{2}={C}_{1}\left(h\nu -{E}_{gd}\right)$$

2 $${\left(n\alpha h\nu \right)}^{0.5}={C}_{2}\left(h\nu -{E}_{gi}\right)$$

where ${E}_{gd}$ is the direct bandgap, ${E}_{gi}$ is the indirect bandgap, *n* is the refractive index, *α* is the absorption coefficient, and *C*_1_ and *C*_2_ are constants. The band structure of materials will be determined using both direct and indirect transitions utilizing these formulas.

**Fig. 9:**
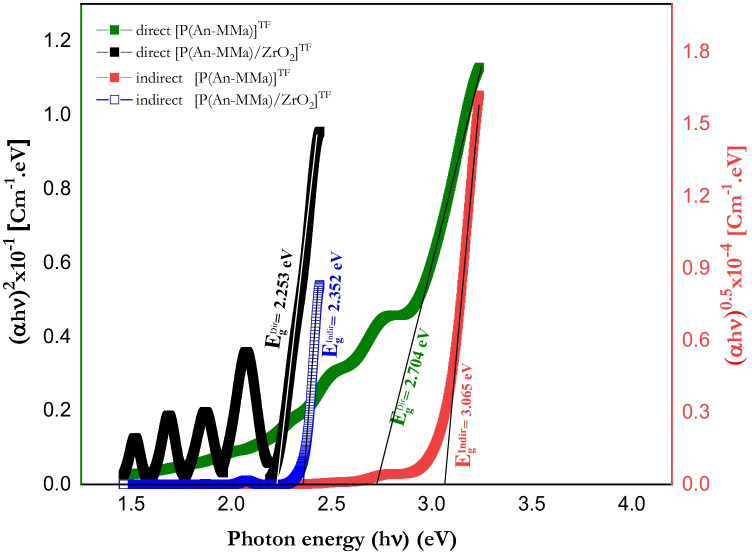
Plot of ${\left(\alpha h\nu \right)}^{0.5}$ and ${\left(\alpha h\nu \right)}^{2}$ vs (*hν*) for the experimental [P(An-MMa)]^TF^ and [P(An-MMa)/ZrO_2_]^TF^ thin film

When a direct bandgap exists, the absorption coefficient depends on the energy of the incident photon reported by (Pankove 1971):

3 $$\alpha h\nu =C{\left(h\nu -{E}_{g}\right)}^{0.5}$$

where ${E}_{g}$ is the bandgap, *C* is a constant dependent on the specimen structure, *α* is the absorption coefficient, *ν* is the frequency of the incident light, and *h* is Planck’s constant. Plots of ${\left(\alpha h\nu \right)}^{0.5}$ and ${\left(\alpha h\nu \right)}^{2}$ versus ($h\nu$) of both the [P(An-MMa)]^TF^ copolymer and [P(An-MMa)/ZrO_2_]^TF^ nanofiber composite films of thickness 100 nm near the absorption edge are shown in Fig. 9, where the nature and width of the bandgap can be determined.

The allowed direct transition energies were determined by extrapolating the linear portion of the curves to zero absorption when the optical bandgaps were evaluated from ${\left(\alpha h\nu \right)}^{2}$ vs $h\nu$ plots, while ${\left(\alpha h\nu \right)}^{0.5}$ vs for allowed indirect transition energies (Fig. 9). For direct allowed transitions, the energy gap was 2.704 eV and 2.253 eV for both [P(An-MMa)]^TF^ copolymer and [P(An-MMa)/ZrO_2_]^TF^ nanofiber composite thin film, respectively. In addition, if we consider that the investigated materials have a possibility of indirectly allowed transitions, the energy gap was 3.065 eV and 2.352 eV for both the [P(An-MMa)]^TF^ copolymer and [P(An-MMa)/ZrO_2_]^TF^ nanofiber composite thin film, respectively. From the obtained data, it is clear that in the indirect transitions, which require photon assistance, the absorption coefficient has the following dependence on the photon energy (Tauc 2012):

4 $$\alpha h\nu =A{\left(h\nu -{E}_{g}+{E}_{p}\right)}^{2}+B{\left(h\nu -{E}_{g}-{E}_{p}\right)}^{2}$$

where ${E}_{p}$ is the energy of the photon associated with the transition and *A* and *B* are constants depending on the band structure. Also, the energy gap (i.e., direct and indirect) has been decreased with the loading of the ZrO_2_ nanoparticles to the P(An-MMa)] copolymers. Table 4 summarizes the energy gap values of both copolymer and nanofiber composite.

**Table 4:** The energy bandgap energies of the copolymer and nanofiber composite

Sample type	Bandgap energy (eV)		Absorption edge (eV) based on Fig. 7
	Direct	Indirect	
[P(An-MMa)]^TF^ copolymer	2.704	3.065	2.755
[P(An-MMa)/ZrO_2_]^TF^ nanofiber composite	2.253	2.352	2.254

**Fig. 10:**
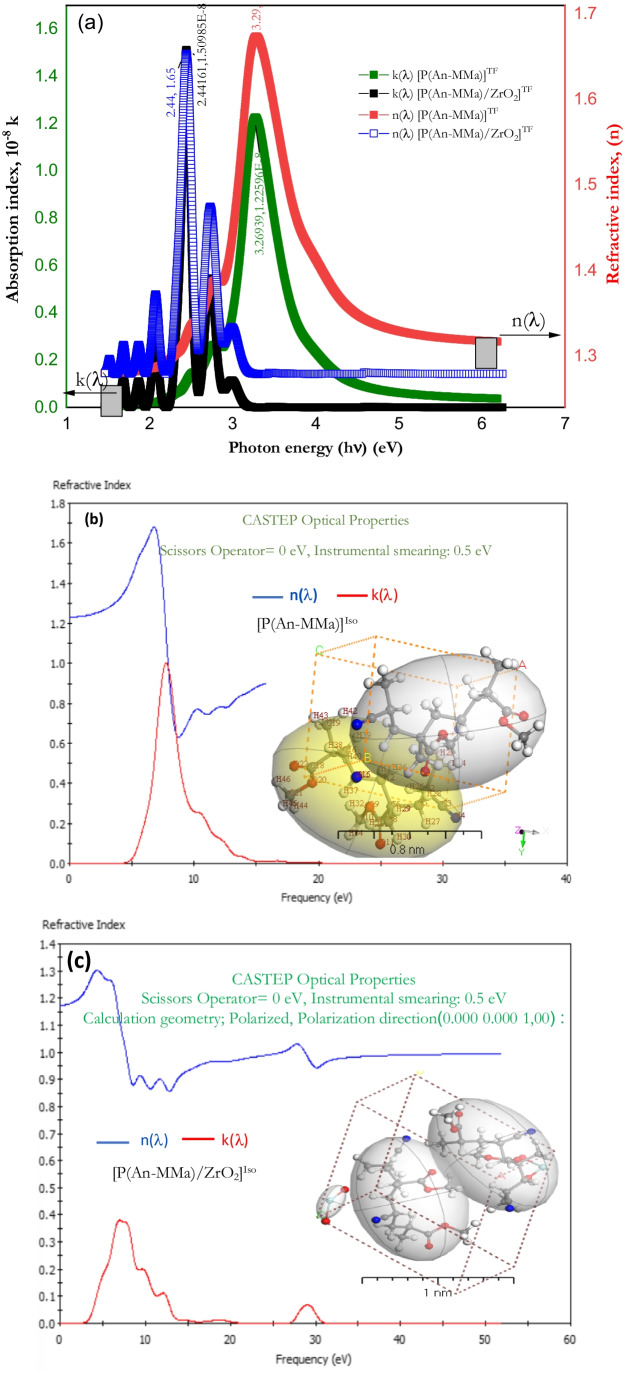
**a** The spectral dependence of both the absorption index (*k*) and the refractive index (*n*) as a function of the photon energy ($h\nu$) for P(An-MMa)]^TF^ copolymer and [P(An-MMa)/ZrO_2_]^TF^ nanofiber composite thin films(experimental). (**b**, **c**) Simulated values of *n* and *k* for both copolymer and nanofiber composite isolated molecules using DFT/CASTEP optical properties

Any optoelectronic or optical sensor device based on copolymers or nanofiber composite materials relies strongly on its dispersion characteristics. So, the obtained data of both refractive (*n*) and absorption (*k*) indices and and below the inter-band absorption edge other dispersion parameters corresponding to the fundamental electronic excitation spectrum are so valuable. A complete characterization of the optical properties of any polymer/copolymer or nanofiber composite materials can be obtained from the complex refractive index ($\widehat{n}$):

5 $$\widehat{n}=n+ik$$

6 $$k=\frac{\alpha \lambda }{4\pi }$$

7 $$n=\frac{1+R}{1-R}+\sqrt{\left[\frac{4R}{{\left(1-R\right)}^{2}}\right]-{k}^{2}}$$

where the real part (*n*) represents phase velocity and is connected to dispersion, while the imaginary part (*k*) represents the mass attenuation coefficient and offers a measure of the electromagnetic wave’s dissipation rate in the dielectric medium. $\lambda$ is the wavelength of the incident photon, $\alpha$ is the absorption coefficient, and *R* is the reflectance. The obtained results of both *n* and *k* for [P(An-MMa)]^TF^ copolymer and [P(An-MMa)/ZrO_2_]^TF^ nanofiber composite film of thickness 100 nm are shown in Fig. 10. Figure 10a represents the experimental results of both *n* and *k* of the thin films at room temperature over the photon energy range of 1.5–6.2 eV (i.e., wavelength from 200 to 850 nm), where throughout this spectral range, an abnormal dispersion spectrum has been obtained which can be explained by the multi-oscillator model (Stendal et al. 1996). For the refractive index (*n*), the [P(An-MMa)]^TF^ copolymer has a broadband peak (at $h\nu =3.29 \mathrm{eV}$) while that for the [P(An-MMa)/ZrO_2_]^TF^ nanofiber composite film is characterized by multi-peaks located at different photon energies. Many unique resonant frequencies (i.e., multi-oscillators) have been obtained for the P(An-MMa)/ZrO_2_]^TF^ film. The oscillations of the bound electrons of the atoms in the visible region and/or the lattice vibrations in the near-infrared region are responsible for the resonances. In addition, the nanofiber composite film has many electronic oscillators of different frequencies, because the total polarization in that region is proportional to the dielectric constant, so, an anomalous dispersion of the refractive index rises in the absorbing region due to electronic transitions between two atomic states. (Fox 2002). On the other hand, the absorption index (*k*) for both the [P(An-MMa)]^TF^ copolymer and [P(An-MMa)/ZrO_2_]^TF^ nanofiber composite has a behavior similar to that of their absorbance spectrum, where the absorption index of the [P(An-MMa)]^TF^ copolymer is characterized by a main broad peak with a maximum value at a photon energy of 3.269 eV, while that of the [P(An-MMa)/ZrO_2_]^TF^ nanofiber composite film has multi-peaks at photon energy ranging from 1.4 to 3.2 eV. The absorption index spectra of [P(An-MMa)/ZrO_2_]^TF^ nanofiber composite films exhibit two main absorption peaks in the visible region (447.59 and 508.13 nm) and four secondary peaks in the visible and near-infrared regions. The absorption peaks are attributed to the electronic transitions across π–π* orbits. Also, Fig. 10b, c show the estimation of both the refractive index (*n*($\lambda$)) and the absorption index (*k*(*λ*)) by using DFT simulation of the [P(An-MMa)]^TF^ copolymer and [P(An-MMa)/ZrO_2_]^TF^ nanofiber composite isolated composite molecule. The simulated values are very similar to the DMol^3^ geometry optimization model values obtained via DFT compared with the experimental values.

The increase in electrical conductivity is caused by the delocalization of electrons that spread throughout the material. Conductivity is also increased by the movement of charge carriers such as ions and electrons in the crystal lattice, which is produced by incident electromagnetic waves. The optical response to any investigated materials such as copolymers or nanofiber composites could be determined by its optical conductivity $\sigma (\lambda )$, where $\sigma (\lambda )$ is given by the following equation (Leclerc et al. 1993):

8 $$\sigma \left(\omega \right)= {\sigma }_{1}\left(\omega \right)+i{\sigma }_{2}\left(\omega \right)$$

where ${\sigma }_{1}\left(\omega \right)$ and ${\sigma }_{2}\left(\omega \right)$ represent the real and imaginary parts of the optical conductivity. Also:

9 $$\begin{array}{ccc}{\sigma }_{1}\left(\omega \right)= \omega {\varepsilon }_{2}{\varepsilon }_{0}& \mathrm{and}& {\sigma }_{2}\left(\omega \right)=\omega {\varepsilon }_{1}{\varepsilon }_{0}\end{array}$$

10 $$\begin{array}{ccc}{\varepsilon }_{1}= {n}^{2}-{k}^{2}& \mathrm{and}& {\varepsilon }_{2}=2nk\end{array}$$

${\varepsilon }_{1}$ and ${\varepsilon }_{2}$ are the real and imaginary parts of the optical dielectric constants of the materials. Finally, *ω* is the angular frequency, and ${\varepsilon }_{0}$ the permittivity of free space.

**Fig. 11:**
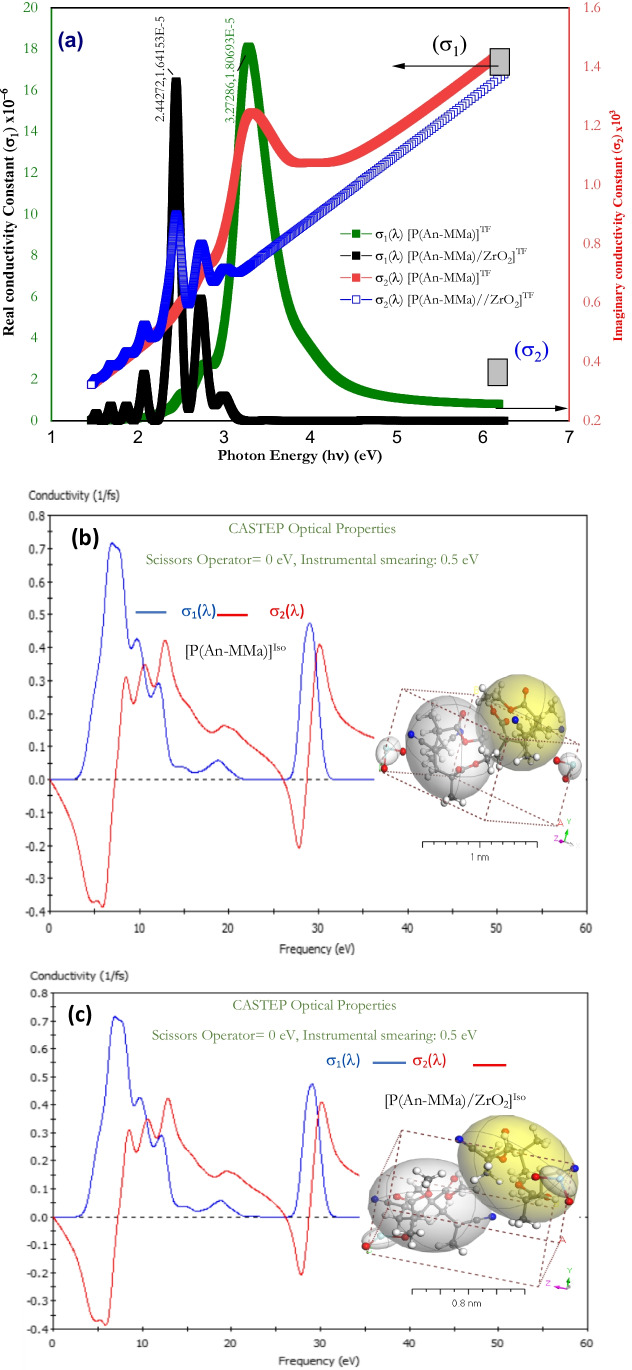
**a** ${\sigma }_{1}$ real and ${\sigma }_{2}$ imaginary optical conductivities of the P(An-MMa)]^TF^ copolymer and [P(An-MMa)/ZrO_2_]^TF^ nanofiber composite thin films as a function of the photon energy (experimental). (**b**, **c**) Simulation of ${\sigma }_{1}$ and ${\sigma }_{2}$ using CASTEP optical properties of both the copolymer and nanofiber composite

Figure 11a shows the dependence of both ${\sigma }_{1}\left(\omega \right)$ and ${\sigma }_{2}\left(\omega \right)$ optical conductivities of the P(An-MMa)]^TF^ copolymer and [P(An-MMa)/ZrO_2_]^TF^ nanofiber composite thin films with the photon energy of range from 1.2 to 6.4 eV. It is observed that the real part of the optical conductivity ${\sigma }_{1}\left(\omega \right)$ of both copolymer and nanofiber composite films has the same pattern like the refractive index of each one. A broadband peak characterizes ${\sigma }_{1}\left(\omega \right)$ of the P(An-MMa)]^TF^ copolymer film. It has a maximum value at $h\nu =3.2729 \mathrm{eV}$ (corresponding to the maximum absorption peak band value). Likewise, for the [P(An-MMa)/ZrO_2_]^TF^ nanofiber composite thin film, multiple peaks in the photon energy range from 1.4 to 3.2 eV have been obtained. The main peak is located at 2.4427 eV. ${\sigma }_{1}\left(\omega \right)$ increased with photon energy, especially at the absorption band in both the copolymer and nanofiber composite, which may be assigned to the excitation of electrons in this region, or probably due to the high absorbance coefficient related to the presence of the thin film, with a charge ordering effect. where the absorption coefficient ($\alpha$) and optical conductivity are linked by the following expression (Zuo et al. 1987):

11 $$\sigma =n\frac{\alpha c}{4\pi }$$

where *c* is the velocity of light. On the other hand, $${\sigma }_{2}\left(\omega \right)$$, the imaginary part of the optical conductivity of the P(An-MMa)]^TF^ copolymer and [P(An-MMa)/ZrO_2_]^TF^ nanofiber composite thin films, has patterns unlike those of $${\sigma }_{1}\left(\omega \right)$$ in the photon energy range from 1.2 to 6.4 eV (Fig. 11a). $${\sigma }_{2}\left(\omega \right)$$ is linearly increased with photon energy with a maximum band broad peak at 3.2729 eV for the copolymer and a sharp narrow peak at 2.4427 eV for the nanofiber composite. Also, $${\sigma }_{2}\left(\omega \right)$$ is characterized by its so high values related to that of $${\sigma }_{1}\left(\omega \right)$$ (due to its dependency on $${n}^{2})$$.

Figure 11b and c demonstrate the simulation of both ${\sigma }_{1}$ and ${\sigma }_{2}$ of the P(An-MMa)]^TF^ copolymer and [P(An-MMa)/ZrO_2_]^TF^ nanofiber composite thin films by using DFT/CASTEP optical properties. From the figure, it is clear that there is a good agreement between experimental and simulation results.

### DC electrical properties of [P(An-MMa)/ZrO_2_]^TF^ thin film

**Fig. 12:**
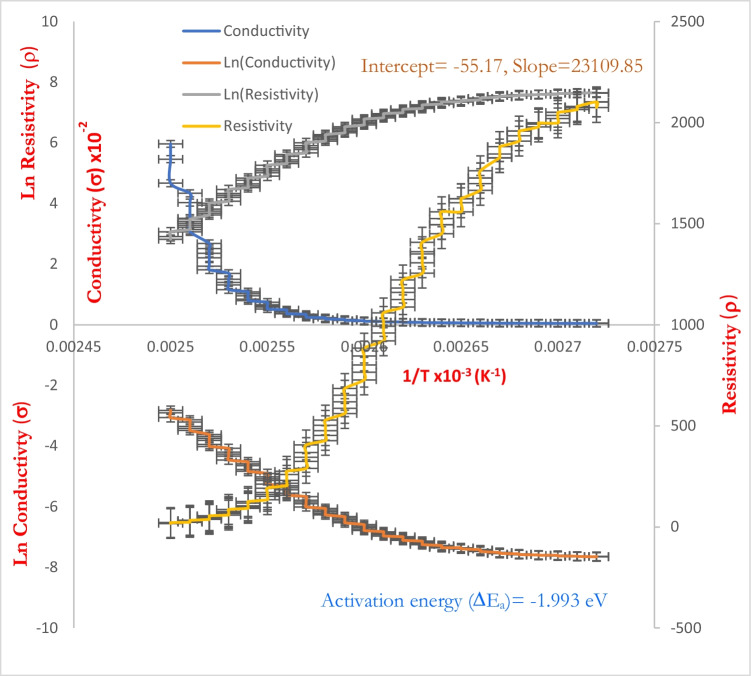
Dependence of the DC conductivity/resistivity on 1/*T* of the [P(An-MMa)/ZrO_2_]^TF^ nanofiber composite thin film

The magnitude and temperature dependence of the DC electrical conductivity or resistivity is one of the most important measurements made by those investigating newly fabricated materials such as copolymers or nanofiber composites. Such results can be utilized to compute the activation energy, $\Delta {E}_{g}$, for the electrical conduction process in the materials under investigation, as well as to assess the degree of polymerization. The magnitude and temperature dependency of a copolymer’s or nanofiber composite’s electrical resistivity or conductivity is a function of the material’s molecular structure, the nature and number of current carriers, and the temperature. The conductivity of any semiconductor material increases as the temperature rises. This may be due to an increase in charge transfer efficacy (Chougule et al. 2011). The curling of the copolymer chain was also a result of thermal transfer. This phenomenon enhances the electrical conductivity of the polymer backbone by stressing the conjugation within it (Kobayashi et al. 1993). Figure 12 shows the variation of the DC conductivity/resistivity of the [P(An-MMa)/ZrO_2_]^TF^ nanofiber composite thin film. Also, the relation of $\mathrm{ln}{\sigma }_{dc}$ and $\mathrm{ln}{\rho }_{dc}$ vs 1/T has been carried out, where the electrical conductivity of the [P(An-MMa)/ZrO_2_]^TF^ nanofiber composite film was performed in the temperature range of 350 and 400 K. It is clear that electrical conduction in the [P(An-MMa)/ZrO_2_]^TF^ nanofiber composite thin films is dominated by polarons and bipolarons. The activation energy, $\Delta {E}_{a}$, of the thin film could be calculated from the linear curve fitting of the relation between $\mathrm{ln}\sigma$ and $\frac{1}{T}$ according to the Arrhenius equation (Gosh et al. 1999):

12 $${\sigma }_{dc}= \frac{{A}_{0}}{T}{\mathrm{exp}}^{\left({~}^{-\Delta {E}_{a}}\!\left/ \!{~}_{kT}\right.\right)}$$

where *A*_o_ and $\Delta {E}_{a}$ represent the pre-exponential factor and activation energy, respectively, and *k* is Boltzmann’s constant (*k* ≈ 8.617 10^−5^ eV/K). From Fig. 12, it is clear that both $\rho$ and $\sigma$ are strongly dependent on temperatures. Also, we can consider two temperature regions: the first (380–400 K) and the second (366.97–380 K); there are two activation energies in the nanofiber composite thin film. The activation energy of the first region is 1.993 eV and 0.423 eV for the second. Both first and second activation energies occur at high temperature, and the thermal excitation conduction mechanism of charge carriers from grain boundaries to a neutral region of the material is responsible for the polymer’s electrical conductivity (first region) (Zhao et al. 2002), and may be due to the charge carriers’ transport (hopping) (i.e., Mott’s variable range hopping, VRH) to localized states near the conduction band (Fermi level) of the nanofiber composite film (second region). In addition, Fig. 12 shows the DC resistivity as a function of 1/*T* (K^−1^) for the [P(An-MMa)/ZrO_2_]^TF^ nanofiber composite film. Starting at 350 K, the resistivity of samples drops exponentially with increasing temperature, demonstrating the usual semiconductor behavior of nanofiber composites, according to the Verwey hopping model. It can be inferred that the [P(An-MMa)/ZrO_2_]^TF^ nanofiber composite film showed semiconductor properties. It is mainly due to the thermally activated mobility of the carriers (electrons or holes) rather than the thermally activated generation of the carriers (Nurhayati et al. 2021). The [P(An-MMa)/ZrO_2_]^TF^ nanofiber composite film was found to have lower resistivity values compared with the individual polymers or pure phase ZrO_2_ nanofiber particles from room temperature to 400 K. These phenomena may be because ZrO_2_ contributes to the charge carriers to the matrix and there is no scattering of charge carriers.

From plotted data of Fig. 12 and Table 5, the value of activation energy $(\Delta {E}_{a})$ is the minimum value of energy needed to resolve potential system barriers and was determined based on the slope of the straight lines. Finally, the $\Delta {E}_{a}$ (eV) values decreased from [ZrO_2_]^NPs^ to [P(An-MMa)/ZrO_2_]^TF^ nanofiber composite resulting from the [ZrO_2_]^NPs^ dispersion in the matrix of [P(An-MMa)]^TF^ adding more conducting directions, which resulted in higher $({\sigma }_{dc})$ values and lower $\Delta {E}_{a}$(eV) values, which were in line with the crystallization degree demonstrated by the XRD measurements. The results presented in Table 5 designate that the activation energy is influenced by the type of polymer and type of formed junction (homojunction and heterojunction). The field-dependent nature of high series resistance of the organic layer could be the essential reason for this effect. Given the small temperature range of kinetic studies, the activation energy can accurately be approximated as temperature independent, the activation energy being an indication of the speed of the crystallization rate, thermal stability, and glass transition temperature (*Tg*). It can be concluded that the activation energy of [P(An-MMa)/ZrO_2_]^TF^ is $\Delta {E}_{a}=1.993 \mathrm{eV}$ (highest activation energy) for that is the lowest rate of crystallization with the highest thermal stability in the second stage [83,86], while for the lowest value of activation energy in the first stage, it can be concluded that [ZrO_2_]^NPs^ has the highest rate of crystallization and the smallest thermal stability. The resulting data of DrTGA thermal analysis (Fig. 3) indicate that ${T}_{g}$ is known to be a strong indicator of the thermal stability of the composite. The values of ${T}_{g}$ for all the samples are given in Table 5. The highest ${T}_{g}=438.83 ^\circ \mathrm{C}$ for [P(An-MMa)/ZrO_2_]^TF^, which corresponds to the highest thermal stability and activation energy.

**Table 5:** Activation energy $(\Delta {E}_{a})$ values for the [P(An-MMa)/ZrO_2_]^TF^ and [ZrO_2_]^NPs^ films and composition with a different polymer

Film composition	Symbols	$\Delta {E}_{a1}$ (eV)	$\Delta {E}_{a2}$ (eV)	$({T}_{g})$	References
		Stage 1	Stage 2		
Zirconium oxide nanoparticles	[ZrO_2_]^NPs^	0.124	0.376	-	Mahmoud et al. (2020)
Poly(o-phenylenediamine) + poly(p-toluidine)	[PoDA + PpT]^TF^	0.263	0.907	4.17	
Poly(o-phenylenediamine) + poly(p-toluidine)/zirconium oxide nanoparticles	[PoDA + PpT/ZrO_2_]^C^	0.002	1.170	55.13	
Polyethylene oxide/carboxymethylcellulose	PEO (10%)/CMC (90%)	-	10.42	536.25	Rajeh et al. (2019)
	PEO (10%)/CMC (90%)	-	11.47	-	
Poly(acrylonitrile-co-methylmethacrylate)/zirconium oxide nanoparticles	[P(An-MMa)/ZrO_2_]^TF^	-	1.993	439.83	Present work

## Conclusion

In this work, a powder form of poly P(An-MMa) polymer was first synthesized by precipitation polymerization. Also, the powder form of the new nanofiber composite of P(An-MMa) with zirconium dioxide nanoparticle [ZrO_2_]^NPs^ was synthesized using the sol–gel method. Thin films of thickness 100 nm of both the [P(An-MMa)]^TF^ copolymer and [P(An-MMa)/ZrO_2_]^TF^ nanofiber composite via the physical vapor deposition (PVD) technique at room temperature were fabricated. The thickness of the fabricated [P(An-MMa)/ZrO_2_]^TF^ thin film is 98.30 nm utilizing a quartz crystal microbalance (UNIVEX 250 Leybold machine). Raman spectroscopy, TGA, and XRD were used to investigate the structural properties. Raman data show that peaks exhibit a minor change in the band’s peak position, which may result from secondary bonding in nanofiber composites between [P(An-co-MMa)] and [ZrO_2_]^NPs^. The average crystallite size of [P(An-MMa)/ZrO_2_]^TF^, as determined by XRD calculations, is 171.04 nm. The TD-DFT calculations accurately matched the observed XRD and Raman spectra and validated the molecular structure of the examined materials. The optical energy bandgaps (direct and indirect) computed experimentally for [P(An-MMa)/ZrO_2_]^TF^ are 2.253 and 2.352 eV, compared to 2.704 and 3.065 eV for the copolymer film, respectively. The bandgap energy of the pristine copolymer can be reduced by adding [ZrO_2_]^NPs^ to the copolymer blend; this reduced the bandgap energy by a factor of 44.39%, whereas the isolated molecule of the composite [P(An-MMa)/ZrO_2_]^Iso^ has a bandgap of 2.415 eV as determined by TD-DFT/DMol^3^. The stability of the fabricated materials can be verified theoretically by estimating the values of ${E}_{HOMO}$ and ${E}_{LUMO}$ energies; negative values indicate the stability of the products. The DC conductivity characteristics of the copolymer nanocomposite can be improved by adding [ZrO_2_]^NPs^ nanoparticles due to the enhancement of charge transfer caused by ZrO_2_ nanoparticles. [P(An-MMa)/ZrO_2_]^TF^ can be utilized in fabricating Au/[An + PNAn/ZrO_2_]^NC^/n-Si/Al polymer solar cells.

## Data Availability

The data presented in this study are available in the article.
